# Conducting health services research during the COVID-19 pandemic: experiences from the veterans health administration

**DOI:** 10.1186/s12913-023-10296-y

**Published:** 2023-11-16

**Authors:** Brigid Connelly, Edward Hess, Marguerite Daus, Catherine Battaglia, Heather M. Gilmartin

**Affiliations:** 1 Denver/Seattle Center of Innovation for Veteran-Centered and Value Driven Care, VA Eastern Colorado Healthcare System, 1700 N. Wheeling St, Aurora, CO 80045 USA; 2 Health Systems, Management and Policy, University of Colorado, School of Public Health, Aurora, CO 80045 USA

**Keywords:** Health services research, COVID-19, Workforce, Telework, Veterans

## Abstract

**Background:**

Health services researchers within the Veterans Health Administration (VA) seek to improve the delivery of care to the Veteran population, whose medical needs often differ from the general population. The COVID-19 pandemic and restricted access to medical centers and offices forced VA researchers and staff to transition to remote work. This study aimed to characterize the work experience of health service researchers during the COVID-19 pandemic.

**Methods:**

A REDCap survey developed from the management literature was distributed in July 2020 to 800 HSR&D researchers and staff affiliated with VA Centers of Innovation. We requested recipients to forward the survey to VA colleagues. Descriptive analyses and logistic regression modeling were conducted on multiple choice and Likert scaled items. Manifest content analysis was conducted on open-text responses.

**Results:**

Responses were received from 473 researchers and staff from 37 VA Medical Centers. About half (48%; *n* = 228) of VA HSR&D researchers and staff who responded to the survey experienced some interference with their research due to the COVID-19 pandemic, yet 55% (*n* = 260) reported their programs of research did not slow or stop. Clinician investigators reported significantly greater odds of interference than non-clinician investigators and support staff. The most common barriers to working remotely were loss of face-to-face interactions with colleagues (56%; *n* = 263) and absence of daily routines (25%; *n* = 118). Strategies teams used to address COVID-19 related remote work challenges included videoconferencing (79%; *n* = 375), virtual get-togethers (48%; *n* = 225), altered timelines (42%; *n* = 199), daily email updates (30%; *n* = 143) and virtual team huddles (16%; *n* = 74). Pre-pandemic VA information technology structures along with systems created to support multidisciplinary research teams working across a national healthcare system maintained and enhanced staff engagement and well-being.

**Conclusions:**

This study identifies how the VA structures and systems put in place prior to the COVID-19 pandemic to support a dispersed workforce enabled the continuation of vital scientific research, staff engagement and well-being during a global pandemic. These findings can inform remote work policies and practices for researchers during the current and future crises.

**Supplementary Information:**

The online version contains supplementary material available at 10.1186/s12913-023-10296-y.

## Introduction

The Veterans Health Administration (VA) provides healthcare to over six million veterans each year, education and training for health professions students and residents, and research focused on enhancing the well-being of veterans and the nation through discovery and innovation. The VA Health Services Research & Development (HSR&D) service is one of four research funding branches of the VA Office of Research and Development (ORD) [1]. Health services research is a multidisciplinary field of inquiry that examines access to, and the use, costs, quality, delivery, organization, financing, and outcomes of healthcare services [2]. Health services research is performed by clinicians (e.g., physicians, nurses, psychologists, dentists, social workers, pharmacists), economists, engineers, biostatisticians, and other social scientists (e.g., sociologists, anthropologists, political scientists). The goal of health services research is to produce new knowledge about the structure, processes, and effects of health services for individuals and populations [2].

The majority of VA HSR&D researchers and staff are affiliated with 18 Centers of Innovation (COINs) which address particular clinical priorities (e.g. pain, access, suicide prevention) [3]. HSR&D researchers and staff are embedded within the VA healthcare system. In partnership with clinicians and healthcare leaders, they identify opportunities for improvement, formulate research questions, test interventions, evaluate the costs and impacts of major initiatives, and spread and scale up innovations that address health system priorities [1]. VA HSR&D researchers and staff have been an essential component of the VA’s research program for over three decades [4] .

The unprecedented Coronavirus disease 2019 (COVID-19) pandemic led to significant shifts in U.S. healthcare. This resulted in an increased reliance on research to offer public health solutions to the COVID-19 crisis [5]. During the first months of the pandemic, access to patients, resources, offices, and laboratories were limited. From March to June 2020, ORD instituted an administrative hold on non-critical, in-person interactions or interventions with human subjects (e.g., patients, caregivers, employees) to decrease the risk of virus transmission. This enabled VA facilities to prioritize the handling of COVID-19 cases and their prevention [6]. Around the same time, individual States issued public health stay-at-home orders for non-essential workers to reduce the risk of infection to society [7]. VA HSR&D researchers and staff were requested to continue their work remotely, while clinician investigators were requested to engage in additional clinical activities. Non-clinician investigators and staff were recruited to support clinical activities at VA medical centers [6]. The impact of these actions on the continuation of health services research and the productivity and well-being of VA HSR&D researchers and staff are unknown. The aim of this study was to characterize the experience of VA HSR&D researchers and staff during the first months of the COVID-19 response to inform policies and practices in the current and future crises.

## Methods

This study was a cross-sectional, convenience sample design conducted within the VA HSR&D Service. This study employed an explanatory sequential mixed methods research design (QUANTATIVE ➜ qualitative = explanation), which included a quantitative survey with qualitative open text items [8]. Survey invitations and information about the study were sent to 800 health services researchers on July 24^th^, 2020 and remained open for a duration of two weeks, ending data collection on August 7^th^, 2020. Participants were identified through a national administrative research database (VA ART) that supports reporting and administrative processes for VA HSR&D. Inclusion criteria included affiliation with one of the 18 VA COINs and previous receipt of VA HSR&D research funding. The invitation to participate included a request to forward the survey invitation to “members of your team”. Over the following two weeks, four invitations to participate with a VA REDCap survey link were emailed to participants (Appendix 1). The study was promoted through VA HSR&D social media accounts.

### Survey

We developed a survey informed by the remote work literature [9–11], experiences posted to Twitter (#remote work; #WFH) in the early days of the COVID-19 stay-at-home orders, and the authors’ personal experiences with remote work. The survey was field tested with members of the Colorado Clinical and Translational Sciences Institute to establish face and content validity [12]. The survey captured the following respondent demographics: age, race, ethnicity, gender, educational and professional degree, research role, and VA medical center [13]. Previous remote work experiences were queried using an open text item. Participants rated the extent to which remote work during the COVID-19 pandemic interfered with their research activities (i.e., does not interfere, interferes somewhat, interferes to a great extent), and selected from a list of common barriers to remote work, and the frequency of these barriers. Open text items were available to report additional barriers and workarounds to address barriers to remote work.

Respondents were asked if they would be stopping any research during the COVID-19 pandemic (i.e., none, some, all, not applicable) and were given an option to describe the research put on hold and why. Respondents were asked to select from a list of strategies being implemented by department level leadership, investigators, project leads, or project managers to engage staff in a productive way. Finally, participants were asked to share how they were doing, including how the COVID-19 pandemic was impacting them, how they were adapting and coping, and any short- or long-term concerns.

### Statistical analyses

Survey data were exported from VA REDCap. Data prep and analyses were conducted using R version 3.5.3. for the quantitative data and ATLAS.ti Scientific Software Development GmbH for the qualitative data. The data were stratified by the extent remote work during the COVID-19 pandemic interfered with research activities. We used logistic regression models to examine the association between i) reporting interference with research activities (due to COVID-19 remote work) and covariates of interest (i.e., age, research role, race, prior remote workdays, and gender), and ii) reporting work stoppage of research and the covariates. The top two categories for interference (“Somewhat” and “To a Great Extent”) and for work stoppage (i.e., “Some” and “All”) were collapsed into a single category and were modeled against the “None” category in each case. Age was modeled as a continuous variable (natural spline with 2 degrees of freedom) initially, and then supplementally as a five-category variable (< 34, 34–39, 40–46, 47–55, and > 55 years of age). Missing values for age were imputed as the median age [14]. Role, stage, prior remote workdays, race, ethnicity, and gender variables were all modeled categorically. Regression modeling of work stoppage excluded cases where the response variable was missing (resulting in *N* = 419 for this analysis instead of *N* = 473).

Qualitative responses were analyzed using manifest content analysis [15]. A structured matrix was developed to code the data based on the survey questions. All the text responses were reviewed for content and correspondence for the following questions: other barriers to remote work, reasons for stopping research, workarounds, impact, adaptations, and coping.

## Results

Survey responses were obtained from 473 researchers and staff from 37 VA Medical Centers (range 1–42 responses per center) (Appendix 2); 32% (*n* = 151) of responses were from the VA ART reporter invite list, while 68% (*n* = 321) were forwarded from someone on the invite list. Respondents were primarily female (*n* = 359; 76%), white (*n* = 392, 83%), 44 years old (range:19–75 years), with a PhD (*n* = 188; 40%) or bachelor’s degree (*n* = 137, 29%). Respondents included support staff, (i.e., methodologists, project managers, research administration, research assistant, clinical research role [nurse, social worker, etc.]) (*n* = 270, 57%), clinician investigators (*n* = 96; 20%), and non-clinician investigators (*n* = 91, 19%) (Table 1). Responses to the open-text option for each survey item varied and all thematically mapped to the overarching survey questions and additional themes were identified regarding barriers to remote work and personal and emotional impacts of COVID-19 telework. These themes are noted in the paragraphs below.

**Table 1:** Respondent demographics (*n* = 473)

	**Mean (SD)**
**Age**	43.7 (12.2)
**Gender**	**N (%)**
Female	359 (76)
Male	100 (21)
Non-binary	7 (1.5)
Prefer not to Answer	6 (1.3)
**Race**	
White	392 (83)
Asian or Pacific Islander	34 (7)
Black	18 (4)
Other or did not answer	19 (4)
Multi-racial	10 (2)
**Ethnicity**	
Not of Hispanic Origin	439 (94)
**Educational Degree**	
Doctor of Philosophy	188 (40)
Bachelor of Arts and/or Sciences	137 (29)
Master of Public Health, Nursing, or Social Work	133 (28)
Other	118 (25)
**Professional Degree**	
Medical Doctor/Doctor of Osteopathy	55 (12)
Registered Nurse	15 (3)
**Research Role**	
Support Staff (project manager, methodologist, administration, research assistant, clinical research role)	270 (57)
Clinician Investigator	96 (20)
Non-clinician Investigator	91 (19)
Fellow	17 (4)

Prior to the COVID-19 pandemic, 83% (*n* = 391) reported rarely (0–1 day/week) working remotely. During the pandemic, 69% (*n* = 324) were working remotely 5 days/week. Half of respondents (52% *n* = 244) indicated telework was not interfering with their research and 55% (*n* = 260) reported they had not stopped any research due to the COVID-19 pandemic (Table 2). Of those participants who provided open text responses, 41% (*n* = 101) reported their research had slowed or stopped due to national and local restrictions on conducting non-COVID related in-person research activities, as well as clinical requirements: *“As an Emergency Medicine physician, my clinical time has increased significantly… It feels like research has had to take a back seat”* (Female, Asian or Pacific Islander, Clinician Investigator, Interferes to a Great Extent)*.* An additional 20% (*n* = 51) reported COVID-19-specific safety concerns for their study population (e.g., veterans, VA staff, etc.) or changes to the feasibility of an intervention: *“…implementation studies paused due to staff overwhelmed at facilities we are working with”* (Female, White, Clinician Investigator, Interferes Somewhat).

**Table 2:** Telework days and interference with research due to the COVID-19 pandemic

	**Prior to remote work**	**During remote work**
	N (%)	N (%)
**Telework days per week**		
0 days	279 (59)	27 (6)
1 day	112 (24)	21 (5)
2 days	34 (7)	13 (3)
3 days	13 (3)	30 (6)
4 days	5 (1)	56 (12)
5 + days	30 (6)	324 (69)
**Interference with Research due to remote work**		
None		244 (52)
Some		192 (41)
Great		36 (7)
**Stopping of Research due to remote work**		
None		260 (55)
Some		150 (32)
All		9 (2)
Not Applicable		54 (11)

Results from the regression model of work stoppage showed no association between work stoppage and any of covariates based on a likelihood ratio test (LRT) assessing the (overall) effect of each covariate. Modeling of interference as the response variable showed a statistically significant association (based on LRT) between interference and age, role, and stage.

Relative to the (referent) clinician investigator category, non-clinician investigators (OR: 0.39 [95% CI 0.21-0.73] *p* = 0.003) and support staff (OR: 0.23 (95% [CI 0.13-0.41], *p* < 0.001) reported lower odds of interference. For age modeled categorically, the referent 40–46 category reported the highest level of interference with the 47–55 (OR: 0.32 [95% CI 0.17-0.61], *p* < 0.001) and > 55 (OR: 0.32 [95% CI 0.16-0.64], *p* = 0.001) year old age groups reporting significantly lower odds of interference. We’ve chosen to exclude interpretation/reporting of clinical and translational stage results as most respondents did not report the stage of their research.

### Barriers to remote work during the COVID-19 pandemic

While many respondents did not slow or stop their research, most (81%; *n* = 385) reported experiencing at least one barrier to remote work (Table 3). The most common barriers were missing face-to-face interactions with colleagues (*n* = 263; 56%): *“I have felt lonely. It took me a long time to get used to working away from the office…But most of all I miss the daily interactions with my coworkers, many of whom are also my friends”* (Female, White, Data Programmer, Does not interfere) and absence of daily routine (*n* = 118, 25%): *“It's difficult to find time for self-care because I am tired after looking at a computer screen all day and the lack of routine to go somewhere to physically work is extremely difficult and under stimulating”* (Female, White, Fellow, Does not interfere). Technology issues were a challenge with participants reporting secure VA internet connection issues (*n* = 109, 23%) and general internet issues (*n* = 105, 22%):* “Brief interruptions in internet that disrupt the VA virtual private network (VPN) are the other main hurdle”* (Female, White, Clinician Investigator, Interferes somewhat);*“Technology has been an ongoing source of stress too—recruitment calls and recording qualitative interviews has become a hodge-podge of solutions and with VA also transitioning from Skype to Microsoft Teams, our current workarounds (which took weeks to figure out, depending on the various teams' needs, technology resources, and institutional review board approvals for different tools) are about to be disrupted again.”* (Female, White, Qualitative Methodologist, Interferes somewhat). Additionally, participants reported limited private workspace at home (*N* = 110, 23%): *“One major challenge is that my home- work environment is not optimized for working-from-home. We have no separate space outside of our bedroom and the living room for working, and with two adults working from home and 7-year-old kid three days of the week, there is sometimes no place to have uninterrupted work or meeting time.”* (Female, White, Project Manager, Interferes somewhat) and barriers to childcare (*N* = 87, 18*%): “Although my children are elementary and middle school, they still require attention throughout the day since they have no structured activities (i.e. no camp, no babysitter) …like many women bearing the burden of childcare my career is slowing down”* (Female, Prefer not to say, Clinician Investigator, Interferes somewhat).

**Table 3:** Barriers to remote work and strategies to engage staff

**Barriers to remote work (check all that apply)** (*n* = 473)	N (%)
Missing daily face-to-face interaction (work/social) with colleagues	263 (56)
Absence of daily routine	118 (25)
Secure VA internet connection issues	109 (23)
Limited private workspace in home	110 (23)
Internet issues	105 (22)
No barriers	104 (22)
Other barriers	88 (19)
Childcare Issues	87 (18)
Inadequate IT equipment in home	77 (16)
Elder care	10 (2)
**What strategies are being implemented by local leadership, investigators, project leads, or project managers to engage staff in a productive way (check all that apply)?** (*N* = 322)	
*Videoconference meetings*	375 (79)
*Altered timelines and project expectations*	199 (42)
*Informal video conference-based gatherings (coffee, lunch, social)*	225 (48)
*Daily COVID email updates*	143 (30)
*Group self-care activities (on-line meditation, knitting, book club)*	149 (32)
*Daily huddles *via* phone or video chats*	74 (16)
*Group text updates*	81 (17)
*Other strategies*	41 (9)
*None of these are implemented in my team(s)*	30 (6)

Analysis conducted on 89 open text responses indicated additional barriers to remote work than those captured by the quantitative findings, including challenges conducting research during the COVID-19 pandemic: *“We have been unable to conduct some group interventions and other face-to-face interactions around data collection, intervention delivery, and implementation for a number of the projects that I work on.”* (Female, White, Non-clinician investigator, Does not interfere). Additional challenges included: *“difficulty reaching coworkers rapidly for assistance (phone unreliable, email slow response)”* (Female, White, Clinician Investigator, Interferes somewhat) and professional impacts of the pandemic on their careers. For example, one respondent shared, *“I am fearful that funding is going to be harder to get in the future especially if my team's productivity is low. While trying to maintain flexibility with everyone's mental health, home issues, and logistics, I am really struggling with setting expectations, modifying deadlines, setting priorities, and providing motivation. While working remotely has some positive sides like no commute, it is incredibly draining due to the high amount of effort required for communication”* (Male, White, Non-clinician investigator, Interferes to a great extent).

Personal and emotional impacts of COVID-19 telework were shared, such as: “*I'm worried about getting sick, but I'm more worried about getting my partner, grandparents, or roommates sick. In that regard, I'm happy to be working at home due to less physical risk, but higher emotional risk.”* (Female, White, Fellow, Does not interfere). Another participant shared, *“I'm dealing with legal issues with the death of my dad and uncle, and major house repairs that take a lot of mental energy and occasionally cause disruption (i.e., too loud to work at home, no electricity). I am also deeply affected by the racial protests and am trying to be actively engaged in conversations and actions around antiracism, but am finding that challenging to do from home, as well as balancing it with concerns about physical distancing”* (Female, White, Qualitative analyst, Interferes somewhat).

Local VA leadership support during the transition to remote work varied, with some reporting a positive perception of leadership during the pandemic: *“I am experiencing a lot of personal and professional growth, and much of that comes from the opportunity to reprioritize what I want to work on, and having a receptive audience in my colleagues and leadership to recognize how much we need to pivot our activities”* (Female, White, Non-Clinician Investigator, Interferes somewhat) and *“Leadership has open office hours; dedicated check-in time built into meetings; lots of communication acknowledging difficulties & advocating for flexibility.”* (Female, White, Non-Clinician Investigator, Interferes somewhat). Others reported negative perceptions of their local leadership during the pandemic: *“I feel disconnected to the department as a whole and from leadership as to what future plans are in place. I would prefer more communication even if it is simply to say we have no updates, you can plan to telework for the next weeks/months. To have zero communication leaves me to wonder and worry about the future.”* (Female, White, Research Assistant, Does not interfere). The codes, definitions and counts for the open text responses are organized by level of interference and presented in Appendix 3.

### Workarounds or strategies to remote work barriers

The primary strategy used to support team engagement, productivity and well-being during COVID-19 remote work was videoconferencing (*n* = 375; 79%). Respondents shared: *“We have been using videoconferencing platforms for meetings, which… offers more social interaction than phone.”* (Male, White, Non-clinician investigator, Does not interfere). Teams reported to altering timelines (*n* = 199, 42%): *“Nothing put on hold, but the timelines are being adjusted to accomplish smaller steps while leadership is busy with additional COVID responsibilities”* (Female, White, Program Manager, Does not interfere). Many reported using regular email updates (*n* = 143, 30%), group text updates (*n* = 81, 17%), and huddles via phone or video chat (*n* = 74, 16%). Additional strategies included informal video-based gatherings (*n* = 225, 48%) and starting group self-care activities (*n* = 149, 32%): *“We share what TV shows we're watching, how we're coping, what we're growing, *etc*. As some of the restrictions have been lifted here, we have had a couple of socially distant in-person gatherings to celebrate team birthdays”* (Female, White, Non-clinician investigator, Does not interfere). The codes, definitions and counts for the open text responses are organized by level of research disruption and presented with representative text responses in Table 3 and Appendix 4.

## Discussion

We conducted a survey of VA HSR&D researchers and staff to characterize their experience of conducing HSR&D research during the first five months of the COVID-19 pandemic. Our results indicated that by June 2020, VA HSR&D researchers were experiencing some levels of interference with conducting their work, but the majority had continued their programs of research. Clinical investigators reported higher level of interference compared to non-clinical investigators. Female participants had a higher survey response rate and provided more qualitative stories than male respondents, but no significant differences were found based on gender. Related studies found a similar proportion of response rates based on gender [12], and the greater amount of qualitative data may be the result of increased burden and gender-specific challenges faced by female respondents [16], who took advantage of the opportunity to share their stories. VA HSR&D researchers and staff reported interference with their work due to limited prior telework experience, inadequate home office set-ups, disrupted work routines, and less social interaction. The primary reason research was stopped was due to the administrative hold on non-critical, in-person interactions or interventions with human subjects from March to June 2020. Participants reported multiple workarounds to keep research studies moving forward and keep staff engaged and productive. They also noted that the robust information technology tools provided by the VA and their previous experiences working within dispersed research teams enabled them to adapt to COVID-19 driven telework.

### VA HSR&D work and environment

The study findings are similar to research conducted in large academic centers during the early weeks of the COVID-19 pandemic. Basic and clinical scientists reported halting laboratory and human subjects studies due to a cessation of participant recruitment and site visits, and an increase in clinical and teaching workload [12, 17–21]. As scientists transitioned to remote work and virtual visits, many encountered technological barriers, inadequate workspace, and limited access to materials that challenged data collection and restricted communication between team members. While health services research often requires interaction with patients, caregivers, or employees, there is much work that can be done remotely to continue investigations into the effects of disease and the delivery of health services for individuals and populations. For example, researchers and implementation specialists of one multi-site VA Quality Enhancement Research Initiative (QUERI) program from the Denver-Seattle COIN shared they immediately moved operations online, and conducted recruitment, trainings, meetings, and site visits through Microsoft Teams. The different experiences between basic and clinical scientists and VA HSR&D researchers and staff highlight the unique focus of the work and environment for conducting health services research.

One of the primary strategies that supported the continuation of VA HSR&D research during the COVID-19 pandemic was VA supported web-based tools such as teleconferencing. The VA Office of Information and Technology (OIT) is a national office long focused on innovation and a mission of digital transformation, striving to represent an example of how an exemplary information technology system can further government agency objectives. Pre-pandemic, OIT operated the Digital VA system for VA employees to receive system outage updates, report an issue, or request new equipment or software [22]. At the start of the pandemic, OIT compressed and accelerated their eight-year deployment plan for tele-critical care in an effort to increase telehealth usage during the COVID-19 pandemic. Pre-pandemic investments into virtual private network remote access, teleconferencing, and employee computing equipment prepared many researchers and staff to log in remotely and continue work immediately following the shift to telework [23]. While some in our sample described early barriers due to IT issues, five months into the pandemic, connectivity issues had largely been resolved due to the support of a robust and prepared information technology infrastructure.

The dispersed nature of the VA HSR&D workforce further prepared researchers and staff for the shift to remote work, as many were already using strategies to communicate, build and maintain relationships with colleagues across the nation, prior to the pandemic. Research is a team sport—it depends on collaboration and discourse between team members and within teams to provide insight, dimension, and complexity to further research questions and reach more robust outcomes. Such an approach requires the coordination of multiple personalities, management policies, and communication systems, and poses a challenge for those unprepared to conduct research in a virtual or dispersed setting [24–26]. Spread nationally across 18 COINs, HSR&D researchers and staff collaborate and conduct research daily across geographical barriers [27]. Even before the COVID-19 pandemic, many used videoconferencing and shared data programs and files across research centers. Some participants indicated local-level challenges as in-person operations shifted to remote, yet like the VA OIT efforts, established structures grounded in team science principles allowed research to essentially return to normal five months into the pandemic.

The loss of social opportunities and missing face-to-face interactions with colleagues remained the most prevalent barrier to remote work for HSR&D researchers during late summer 2020. Many reported the lack of spontaneous, informal interaction during hallway conversations or over the proverbial water cooler decreased information exchange and opportunities for innovation, which in turn may have a lasting impact on future research efforts and the generation of new ideas [28]. While HSR&D’s dispersed workforce structure and information technology preparedness helped participants overcome many other challenges, experiences of social or workplace isolation must be considered as remote, or hybrid work becomes the rule. The business and management literature, sectors with a long history of remote work, suggest autonomy and the amount of trust an employee experiences from management are predictors for social or organizational isolation and burnout. Employees who can decide when, where, and how they work are able to sync their time with colleagues and can access or provide support and socialization to coworkers when necessary [29].

As many sectors shifted to remote work in the early days of the pandemic, some managers and leaders expressed skepticism about the sustainability of teleworking, expressing concern that their remote staff would become unmotivated over time [29]. Such doubt led many managers or leaders to micromanage or closely monitor their staff through constant phone calls, instant messaging, or email. While our data suggests frequent check-ins are helpful, there is a clear distinction between regularly scheduled, anticipated check-ins and spontaneous check-ups that cause employees to feel untrusted and stressed, often diminishing their productivity or investment in their work [29]. In a world where technology like Slack, Zoom, or Microsoft Teams allows for frequent, even constant communication, managers must encourage their employees and staff to step away from the computer and take purposeful breaks from their work. As remote work evolves and expectations change, it is important for managers to acknowledge the stress and anxieties of their employees and respond appropriately. Taking time during staff meetings to ask, “How is this remote or hybrid work environment working for you?” or “Is there anything we can be doing better to support your work?” may elicit specific information that can lead to meaningful changes [29]. It is also important to acknowledge non-work-related concerns that may need to be addressed, including preoccupations related to political and societal change or larger world events. Responding to employee stress or anxiety with affirmation and encouragement not only builds employees’ confidence in their leadership, but it also reflects the trust and confidence experienced by management that allows employees to act with a sense of purpose and focus.

### VA national and local leadership, communication, and support

Communication and interaction from engaged local and national VA HSR&D leadership was instrumental in helping research operations continue. Infrequent or poor communication from leadership plagued many other research settings, impacting the productivity and mental health of staff and preventing work-life balance [19]. VA HSR&D researchers experienced similar anxiety and uncertainty in the early weeks of the pandemic, however, by June 2020, communication from local and national leadership appeared to have calmed the initial panic. The support of local leadership allowed participants to identify solutions such as virtual social hours or resetting expectations and timelines. One HSR&D COIN dedicated 30 min to a weekly virtual community hang-out, introduced one week after the official shift to remote work. This voluntary get-together provided a space for participants to detach from work and connect or share tips for adjusting to the uncertainty of the times and ever-changing societal shifts. In addition, National HSR&D leadership introduced the COVID HSR&D Research Rapid Response Initiative to provide researchers and staff funding to examine the range of impacts of the COVID-19 pandemic and the pandemic response on veterans, employees, and the communities we serve [2, 30]. Many of these researchers had to pause their pre-pandemic research efforts, and this support and opportunity from national leadership allowed their work to continue, leading to new insights and collaborations.

### Future considerations

These findings identify crucial strategies employed by the VA, both prior to and during the pandemic, that supported VA HSR&D researchers as they shifted to remote work. However, barriers related to the restrictions on face-to-face gatherings resulted in social isolation for many. As some research centers continue to work remotely or are trialing hybrid work models, more work is needed to support staff. In-person conferences and meetings provide unique opportunities for networking and sharing of research that had been strained by virtual conferences during the pandemic. Much of the interaction at conferences occurs during coffee or lunch breaks, dinner, or drinks, while poster sessions provide a valuable opportunity for junior researchers to gain exposure [31]. Maximizing opportunities for networking and connection at such events and minimizing the sharing of didactic information that could be delivered via virtual platforms would repurpose the meetings for connection and learning.

Similarly, implementing practices to optimize hybrid onboarding and teambuilding will help new and established staff feel connected. The technology sector and other fields with a historically hybrid work force offer some suggestions around how to bring new people in, work within, and lead hybrid teams. These include daily 1-on-1 check-ins with supervisors and small teams during the first two weeks on the job [30] or implementing a “buddy” system for new hires to act as an informal point of contact besides their manager or supervisor. The buddy system approach could provide new hires with opportunities to meet people outside their immediate team and expose them to novel ideas and other ways of working within their organization [28, 32]. Dedicating time and investing in such practices would ensure high-quality team members are hired and are motivated to remain in their position as they navigate a hybrid work world. The future of remote and hybrid work models is uncertain. Fortunately, VA HSR&D researchers are well-positioned to study the impact of hybrid work on health services research and the populations they serve.

### Limitations

The current study is a cross-sectional design conducted in the United States and represents a snapshot of participants’ experiences during the first five months of the COVID-19 pandemic. Further, our small convenience sample consisted predominantly of White, female doctorate-level respondents and may not be representative of all HSR&D researchers and staff. The research team maintained open dialogue during the analyses and manuscript writing to assess the effect of our experiences as primarily female scientists, new to remote work, and working within the VA healthcare system. Given the unique national and local level leadership and operational models in the VA, these results may not be generalizable to other scientists, but instead serve as a model for organizations performing health services research.

## Conclusions

Our findings reflect a growing body of literature that acknowledges the challenges of remote work during the COVID-19 pandemic, adding crucial perspectives and suggestions from the unique field of health services research. Most of our participants were first-time remote workers, and encountered barriers related to information technology and workspace utilization in the early weeks of the pandemic. However, by June 2020, most participants experienced no work stoppage and little to no interference to their research productivity due to strategies and workarounds they employed, with the support of VA local and national leadership. The structures and leadership support employed by VA HSR&D before and during the pandemic, including a robust information technology system and a dispersed yet highly connected workforce was crucial to the continuation of programs of research. Reports of isolation or lack of social interaction with colleagues remained a prevalent issue and warrant further consideration from leadership. Efforts to increase autonomy and trust in employees, as well as adaptations to the focus of research conferences and onboarding and team-building practices will support not only HSR&D researchers and staff but build a body of evidence to inform research teams that continue to work remotely.

### Supplementary Information

**Additional file 1:** **Appendix 1. **REDCap Survey. **Appendix 2. **Survey Respondents VA Medical Centers Location and Sample Size. **Appendix 3. **Other Barriers to Remote Work: Coded Categories. **Appendix 4. **Workarounds Created to Address Barriers to Remote Work.

## Data Availability

The datasets generated and/or analyzed during the current study are not publicly available, as they are restricted to researchers with a VA affiliation. However, they are available from the corresponding author on reasonable request.

## Abbreviations

COIN: Center of Innovation
COVID-19: Coronavirus disease 2019
HSR&D: Health Services Research & Development
LRT: Likelihood ratio test
ORD: Office of Research and Development
OIT: Office of Information and Technology
QUERI: Quality Enhancement Research Initiative
VA: Veterans Health Administration
VA ART: VA Administrative Research Database
