# Supplementary material for: Conducting health services research during the COVID-19 pandemic: experiences from the veterans health administration
Source: BMC Health Serv Res. 2023 Nov 16;23:1267. doi: 10.1186/s12913-023-10296-y (PMC10655445; doi:10.1186/s12913-023-10296-y)
Supplement: Supplementary file 1 — Additional file 1: Appendix 1. REDCap Survey. Appendix 2. Survey Respondents VA Medical Centers Location and Sample Size. Appendix 3. Other Barriers to Remote Work: Coded Categories. Appendix 4. Workarounds Created to Address Barriers to Remote Work. [file 12913_2023_10296_MOESM1_ESM.docx]

**Appendix 1. REDCap Survey**

**From:** Heather Gilmartin, PhD, NP

**Sent:** (Date)

**Subject:** The Impact of Remote Work for VA Researchers During COVID-19

You are being asked to participate in this research study because you are living and working during the current COVID-19 pandemic and are a VA researcher or staff member who conducts and supports research for veterans.

If you join the study, you will be asked to complete the following survey.

This study is designed to understand how individual VA researchers and staff are conducting remote work during the COVID-19 pandemic. For the purposes of this research study, “COVID-19” will refer to the currently circulating pandemic strain of coronavirus, sometimes also referred to as “SARS-CoV-2”.

Your responses to this survey will help us identify remote work best practice workarounds, communication and engagement strategies and coping mechanisms from VA researchers and staff. The results will be shared as a report and/or toolkit to inform current remote work practices. The data we collect will be used for this study but may also be important for future research. Your data may be used for future research or distributed to other researchers for future study without additional consent.

**Your participation in the survey is completely voluntary. All of your responses will be kept confidential. We will not be collecting any information that could be used to identify you**. You have a choice about being in this study. You do not have to be in this study if you do not want to be. The Colorado Multiple Institutional Review Board has approved this survey (Protocol 20-0892). Possible discomforts or risks include potential discomfort with questions asked. There may be risks the researchers have not thought of. This study is not designed to benefit you directly.

**The survey will take you about 5 minutes to complete**. Please click on the link below to go to the survey (or copy and paste the link into your Internet browser) to begin the survey.

Survey link: (to be created)

If you have questions, you can call Heather Gilmartin at 970-471-5392. You can call and ask questions at any time. You may have question about your rights as someone in this study. If you have questions, you can call the Colorado Multiple Institutional Review Board (the responsible institutional review board). Their number is 303-724-1055.

By accessing and completing the survey, you are agreeing to participate in this research study.

Statement by Person Agreeing to Be in Study:

1. I have read and understand the information provided above: (required)
   1. Yes
   2. No
2. I am currently working remotely (5 days/week) due to COVID-19 stay at home mandates: (required)
   1. Yes
   2. No
3. I agree to participate in this study: (required)
   1. Yes
   2. No

*Must click yes to the above 3 questions to proceed*

1. What is your age? (open text) (required)
2. Professional Credentials (Check all that apply) (required)
   1. PhD
   2. MD
   3. MSW
   4. MPH
   5. MSN
   6. RN
   7. BA
   8. BS
   9. Other: (open text)
3. Gender: (select one) (required)
   1. Male
   2. Female
   3. Non-binary
   4. Prefer not to answer
4. Race: (Check as many as apply) (required)
   1. American Indian; Alaskan Native
   2. Asian or Pacific Islander
   3. Black
   4. White
   5. Other (specify)
   6. Prefer not to answer
5. Ethnicity: (optional)
   1. Hispanic origin
   2. Not of Hispanic origin
   3. Prefer not to say
6. Research Role: (Please indicate the role you spend >50% of your time): (select one) (required)
   1. Clinician investigator
   2. Non-clinician investigator
   3. Qualitative Methodologist
   4. Quantitative Methodologist
   5. Fellow
   6. Clinical research role (e.g., nurse, social worker, etc.)
   7. Data Programmer
   8. Project manager
   9. Research Assistant
   10. Research administration (e.g., grants administration, finance)
   11. Other:
7. Please identify your home VA Medical Center (e.g. Rocky Mountain Regional VA Medical Center) (open text) (required)
8. Please briefly describe two projects that are your primary focus during this remote work time (April-July) and your role in these projects: (open text) (optional)
9. What stage are these projects on the clinical and translational research spectrum? (select one) (optional)
   1. Translation to Natural Animal Models (T0.5)
   2. Translation to Humans (T1)
   3. Translation to Patients (T2)
   4. Translation to Practice (T3)
   5. Translation to Population (T4)
   6. Don’t know
10. Prior to the remote work recommendation for COVID-19, how many days a week did you work from home? (select one) (required)
    1. 0 days
    2. 1 day
    3. 2 days
    4. 3 days
    5. 4 days
    6. 5 days
    7. 6 days - 7 days (work from home Monday-Friday, plus some weekends)
11. To what extent does remote work during COVID-19 interfere with your ability to conduct your research activities? (select one) (required)
    1. Does not interfere
    2. Interferes somewhat
    3. Interferes to a great extent
12. What barriers to remote work are you experiencing? (Check all that apply) (required):
    1. No barriers
    2. Internet issues
    3. Secure VPN connection issues
    4. Inadequate IT equipment in home
    5. Childcare
    6. Elder care
    7. Limited private workspace in home
    8. Missing daily face-to-face interaction (work & social) with colleagues
    9. Absence of daily routine
    10. Other:
13. How frequently do these barriers impact your work? (select one) (required)
    1. Rarely
    2. Once a week
    3. 2-3 times a week
    4. Daily
    5. Not applicable
14. What work arounds have you and/or your team created to address barriers to remote work (open text) (optional)
15. Will you be stopping any research during the COVID-19 pandemic? (select one) (required)
    1. None
    2. Some
    3. All
    4. Not applicable
16. Please describe the research that is being put on hold and why (open text) (optional)
17. What strategies are being implemented by local leadership, investigators, project leads, or project managers to engage staff in a productive way? (Check all that apply) (required)
    1. Daily huddles via phone or video chats
    2. Videoconference meetings
    3. Daily email updates
    4. Group text updates
    5. Informal video conference-based gatherings (coffee, lunch or end of week social time)
    6. Group self-care activities (on-line meditation, knitting, book club, etc.)
    7. Altered timelines and project expectations
    8. None of these are implemented in my team(s)
    9. Other:
18. Who else is in your home during the day and/or night (check all that apply) (required):
    1. Live alone
    2. Spouse/partner
    3. Roommate
    4. Children (*dropdown for number and ages if this box is checked*)
    5. Parents
    6. Pets
    7. Other:
19. Tell your story: Please share how you are doing during the COVID-19 pandemic. You can write as little as a sentence or as much as one page. Please do not include any personally identifying information in your description (e.g. names of individuals, specific locations, etc.). In your story, please consider including information on: (open text) (optional)

- How remote work during the COVID-19 pandemic has impacted you
- How you are adapting to remote work during the COVID-19 pandemic
- How you are coping with remote work during the COVID-19 pandemic
- Your concerns in the short and long term

**Appendix 2. Survey Respondents VA Medical Centers Location and Sample Size**


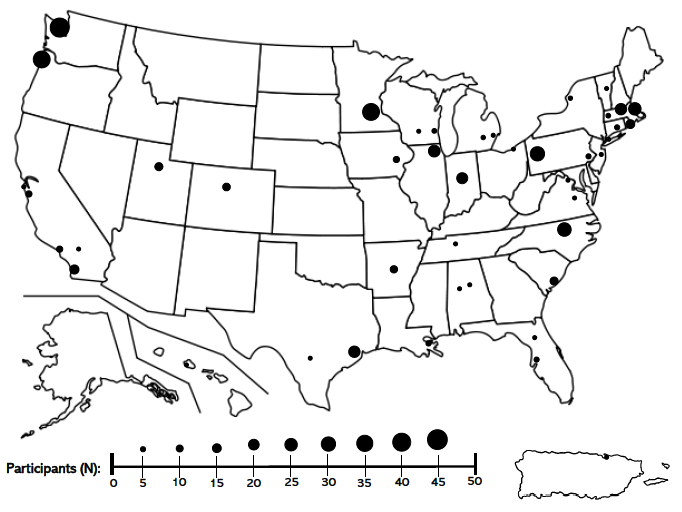


**Appendix 3. Other Barriers to Remote Work: Coded Categories**

| Codes | Definition | Interference Level | Count | Example of Text Responses |
| --- | --- | --- | --- | --- |
| Barrier to working remotely | Barriers related to efficient and/or effective communication, difficulty reaching coworkers, difficulty holding staff accountable, absence of face-to-face interactions, coordinating online meetings | Does Not Interfere | 3 | Barriers are minimal but I do miss the face-to-face interaction with colleagues for building/maintaining connections and rapport |
|  |  | Interferes Somewhat | 7 | difficulty reaching coworkers rapidly for assistance (phone unreliable, email slow response); pulled from research activities to clinical work lacking support |
|  |  | Interferes to a Great Extent | 1 | inability to get meetings |
| Barrier to home office setting | Barriers related to physical setting, equipment, resources (home v. office on campus), disruptions in the home | Does Not Interfere | 16 | Not having a printer available has been at times inconvenient for reviewing manuscripts and double-checking numbers. |
|  |  | Interferes Somewhat | 10 | lack of ergonomic office furniture; cell phone reception is inadequate  Family members thinking I am available when I am working. |
|  |  | Interferes to a Great Extent | 1 | More distractions |
| Barrier to conducting research | Barrier to conduct of research activities (access to subjects, data, materials, collaborators), mailing, research shutdown, implementation studies paused, restriction on human subjects research | Does Not Interfere | 1 | Mailing study materials |
|  |  | Interferes Somewhat | 21 | Limited ability to get organizational context due to inability to conduct in person interviews |
|  |  | Interferes to a Great Extent | 4 | Cannot meet with veterans and staff participants face-to-face for interviews or on-site observations. Study protocols had to be adapted when possible to virtual contact. |
| Impact of pandemic (personal) | Personal pandemic specific issues such as absence of work-life balance, keep up with activities at VA, less exercise | Does Not Interfere | 2 | less connection to activities at the medical center |
|  |  | Interferes Somewhat | 3 | inability to balance out 'work time' from 'non-work time' with restrictions for things to do outside of work (i.e., little work-life balance currently) |
|  |  | Interferes to a Great Extent |  | N/A |
| Impact of pandemic (professional) | Professional pandemic issues such as loss of productivity, difficulty with focus, IRB challenges, difficult to social distance in-office, increase in clinical practice | Does Not Interfere | 0 | N/A |
|  |  | Interferes Somewhat | 4 | zoom fatigue; lower motivation in general |
|  |  | Interferes to a Great Extent | 2 | increased clinical load (emergency medicine)  Lack of focus and organization |
| Non-COVID related challenges | Death in family (non-COVID), major home repairs | Does Not Interfere | 0 | N/A |
|  |  | Interferes Somewhat | 2 | Dealing with death of family members (not COVID related); major house repairs |
|  |  | Interferes to a Great Extent |  | N/A |
| VA Technology barriers | Internet, CAG, lacking or ineffective research software applications accessed through VPN | Does Not Interfere | 13 | working through Citrix is slow and my laptop screen is much smaller |
|  |  | Interferes Somewhat | 10 | Inadequate IT Support from VA and private IT providers  videoconferencing (skype, VANTS) not working and we haven't transitioned to Webex or teams |
|  |  | Interferes to a Great Extent | 0 | N/A |
| Leadership does not support remote work | Wants staff to work on-site, limited support | Does Not Interfere | 1 | Zero support from Research Service right now for everything from hiring to ordering supplies |
|  |  | Interferes Somewhat | 1 | facility leadership wants staff to work on site despite pandemic |
|  |  | Interferes to a Great Extent | 0 | N/A |

**Appendix 4. Workarounds Created to Address Barriers to Remote Work**

| Codes | Definition | Interference with Research Categories | Count | Example of Text Responses |
| --- | --- | --- | --- | --- |
| Increased communication/ meetings | Workaround is increasing and/or establishing routine communication and meetings | None | 22 | *…weekly, not daily, to avoid overtaxing both resources and people* |
|  |  | Some | 7 | *Weekly or monthly huddles* |
|  |  | All | 1 | *Weekly updates from local VAMC leadership* |
| Non-video communication approaches | Communication that occurs over non-video formats | None | 8 | *Increased use of phone calls, phone text messages, IM, email updates* |
|  |  | Some | 6 | *Weekly group email updates* |
|  |  | All | 1 | *Pertinent information emails* |
| Provision of social support | Provision of group-focused social support to address remote work challenges | None | 10 | *Admin sends an inspirational daily quote, which I find very connecting* |
|  |  | Some | 6 | *Created a 'COVID Creativity' e-newsletter so positive photos and stories about our work-from-home lives can be shared with colleagues.* |
|  |  | All | 0 | *N/A* |
| Videoconference meetings | Workaround is use of video conferencing technology | None | 5 | *Video check-ins with investigators and staff* |
|  |  | Some | 0 | *N/A* |
|  |  | All | 1 | *Exploration of VA video platforms* |
| Conduct work that can be done remotely | Workaround is conducting work that can be done remotely | None | 0 | *N/A* |
|  |  | Some | 2 | Pivoting to other data collection |
|  |  | All | 0 | *N/A* |
| Return to normal | Work has returned to pre-COVID normal activities | None | 0 | *N/A* |
|  |  | Some | 3 | *The monthly staff meeting was held every week for a few months but has now returned to 'normal’* |
|  |  | All | 0 | *N/A* |
| Alter data collection method | Alter research methods to remote format | None | 0 | *N/A* |
|  |  | Some | 0 | *N/A* |
|  |  | All | 1 | *Phone interviews with veterans* |
| Use web-based project management tools | Workaround is to use web-based project management tools | None | 1 | *Project tracking via Basecamp* |
|  |  | Some | 0 | *N/A* |
|  |  | All | 0 | *N/A* |
